# A flavone-polysaccharide based prescription attenuates the mitochondrial dysfunction induced by duck hepatitis A virus type 1

**DOI:** 10.1371/journal.pone.0175495

**Published:** 2017-04-10

**Authors:** Hongxu Du, Jingjing Yang, Jingying Bai, Ke Ming, Jintong Shi, Fangke Yao, Wei Zhang, Yang Yu, Yun Chen, Wen Xiong, Yi Wu, Deyun Wang, Yuanliang Hu, Jiaguo Liu

**Affiliations:** Institute of Traditional Chinese Veterinary Medicine, College of Veterinary Medicine, Nanjing Agricultural University, Nanjing, P R China; University of Texas Health Science Center at San Antonio, UNITED STATES

## Abstract

The principal target organ of duck hepatitis A virus type 1 (DHAV-1) is duckling liver, which is an energy-intensive organ and plays important roles in body’s energy metabolism and conversion. As the “power house” of the hepatocytes, mitochondria provide more than 90% of the energy. However, mitochondria are much vulnerable to the oxidative stress for their rich in polyunsaturated fatty acids. Although previous researches have demonstrated that DHAV-1 could induce the oxidative stress in the serum of the infected ducklings, no related study on the mitochondria during the pathological process of DVH has been reported by far. To address this issue, we examined the HE stained tissue pathological slices, detected the hepatic SOD, CAT and GPX activities and MDA contents and analyzed the ATP content, mitochondrial ultrastructure and the mitochondrial SOD, GPX activities and MDA content in the liver tissues. The results showed that the hepatic redox status was significantly disturbed so that causing the mitochondrial dysfunction, ATP depletion and mitochondrial oxidative stress during the process of the DHAV-1 infection, and a prescription formulated with *Hypericum japonicum* flavone, *Radix Rehmanniae Recens* polysaccharide and *Salvia plebeia* flavone (HRS), which had been demonstrated with good anti-oxidative activity in serum, could effectively alleviate the hepatic injury and the oxidative stress in liver tissue induced by DHAV-1 thus alleviating the mitochondrial injury and oxidative stress. In a word, this research discovers the oxidative stress induced mitochondrial dysfunction and oxidative stress during the DVH pathological process and demonstrates HRS exerts good anti-oxidative activity in liver tissue to protect mitochondria against reactive oxygen species (ROS).

## Introduction

Duck viral hepatitis (DVH) is characterized clinically as an acute infection causing high mortality [1]. The pathogen of this disease is duck hepatitis virus (DHV) which mainly infects ducks and do not infect other animals and human beings. Originally, it was divided into three serotypes: serotype 1 (DHV-1), serotype 2 (DHV-2) [2] and serotype 3 (DHV-3) [3]. According to decision of the Virus Taxonomy Ninth Report of the International Committee on Taxonomy of Viruses (ICTV), DHV-1 was classified as a member of *Picornaviridae* family and known as duck hepatitis A virus (DHAV), while DHV-2 and DHV-3 were classed as astroviruses, renamed as duck astrovirus type 1 (DAstV-1) and duck astrovirus type 2 (DAstV-2), respectively. Recently, based on phylogenetic analysis and cross-neutralization tests, DHAV was categorized into three distinct serotypes, the classical serotype (DHAV-1) which formerly known as DHAV, DHAV type 2 (DHAV-2) which was a new serotype isolated in Taiwan [4] and DHAV type 3 (DHAV-3) which was a recently isolated serotype in South Korea [5]. Among these three types, DHAV-1 is deemed to be the most virulent one mainly infecting ducklings within three weeks and causing mortality rate higher than 80% [6]. However, to date, effective therapeutic drugs in the clinic have not been developed, so it still causes a great threat to the poultry farming.

Traditional Chinese medicine (TCM) has a long history of clinical application taking the form of prescription for various viral disease therapies in China and some other Asian counties [7]. With the intensive modern pharmacology study on TCM in the past a period of time, it has attracted increasing attention worldwide [8–10]. For instance, Chen et al. discover Jinxin oral liquid can ameliorate lung inflammation and inhibit virus replication in respiratory syncytial virus-infected mice [11]. Moreover, the research of Liu et al. also suggests that Fuzhenghuayu capsule alleviates liver fibrosis due to chronic hepatitis B without any adverse effect [12]. Here, we also developed a prescription formulated with *Hypericum japonicum* flavone, *Radix Rehmanniae Recens* polysaccharide and *Salvia plebeia* flavone (HRS prescription) with good curative effect against DVH induced by DHAV-1 [13]. However, the underlying mechanisms were still undiscovered.

As the main target organ of DHAV-1, hepatic injury is the most common pathological change in ducklings infected with DHAV-1. Recent researches have demonstrated that oxidative stress contributes as the pathogenesis to this change [13, 14]. Under normal physiological conditions, reactive oxygen species (ROS) are generated and eliminated continuously to keep the dynamic balance [15]. However, once ROS production overwhelms antioxidant defenses, it will result in oxidative stress and causing the molecules and cells injury, even cell death [16]. As one of the most important cellular organelles and the main sites of aerobic respiration, mitochondria play a crucial role in oxidative phosphorylation and most of the cellular energy adenosine triphosphate (ATP) production [17–19]. On one hand, they generate ATP by respiratory chain. On the other hand, ROS are also produced through the way of respiratory chain electronic leakage [15]. However, mitochondria are also the target of the ROS, for they are rich in polyunsaturated fatty acids [20]. Therefore, once mitochondria dysfunction occurs, it will generate a vicious circle of ROS production and aggravate the hepatic injury [21]. What’s more, altered mitochondrial function has been proved to be linked to many kinds of oxidative stress hepatic diseases [22, 23]. However, by far, no related study on the mitochondria during the pathological process of DVH has been reported. Therefore, we hypothesize that mitochondrial dysfunction may be also involved in the pathological process of DVH induced by DHAV-1 and try to explore the underlying hepatoprotective mechanism of the HRS prescription based on this hypothesis.

## Materials and methods

### Ethics statement

This research was performed according to the guidelines of the Institutional Animal Care and Use Committee (IACUC) and Nanjing Agricultural University IACUC and the protocol was approved by the Nanjing Agricultural University IACUC specifically with the project number 2012GGC15003. All efforts were done to reduce the number of ducklings used and their sufferance in the respect of the 3R rule.

### Preparation of drug solution

The preparation process of HRS prescription was the same as our previous study [13]. In short, HRS prescription was prepared based on three ingredients (*Hypericum japonicum* flavone, *Radix Rehmanniae Recens* polysaccharide and *Salvia plebeia* flavone), and the proportion of them in the prescription was 2:1:2 respectively. Then, the prescription was diluted into 3 mg/mL and stored in 4°C. *Hypericum japonicum* flavone and *Salvia plebeia* flavone were purchased from Nanjing Zelang Medical Technology Co., Ltd, and the *Radix Rehmanniae Recens* polysaccharide was prepared and purified in our laboratory.

### Reagents and virus

BCA protein assay kit (detection threshold: 50–1000 μg/mL) was Nanjing Aogene Biotech Co., Ltd product (Nanjing, China). Superoxide dismutase (SOD) assay kit, Catalase (CAT) assay kit, Glutathione peroxidase (GPX) assay kit and Malondialdehyde (MDA) assay kit were purchased from Nanjing Jiancheng Bioengineering Institute (Nanjing, China). ATP assay kit (detection threshold: 5 nmol/L-10 μmol/L) and Tissue mitochondria isolation kit were purchased from Beyotime (Jiangsu, China).

DHAV (*LQ*_*2*_ strain) was obtained from the Shandong Institute of Poultry in China. It was diluted to 10 LD_50_ (2.5 × 10^−1^) with sterile saline solution and used in challenge test right after it was ready [24].

### Animal grouping and treatment

180 one-day-old cherry valley ducklings without inoculated by DHV vaccine were purchased from Chaoyang hatchery (Anhui, China). After acclimating to the new environment for 5 days, the ducklings were randomly divided into 3 groups: Blank control (BC) group (separately reared), Virus control (VC) group and HRS group. For ducklings in VC and HRS groups, they were challenged with 0.2 mL of DHAV-1 solution by intramuscular injection. The ducklings in the BC group were simultaneously injected with an equal volume of physiological saline. Two hours later, ducklings in the HRS group were orally administrated with prepared drug solution by drinking water, once a day for five days. The dosage of the HRS was 3 mg net drug per duckling, and the dosages of these drugs were determined based on our previous research. For the purpose of ensuring consistency across tests, ducklings in the BC and VC groups were orally administrated with the same volume of solvent-added solution. During the entire experiment session, the ducklings were well nursed to reduce all kinds of stress. To minimize the suffering of the ducklings, all efforts were made strictly in accordance with the regulation of animal protection committee.

### Mortality rate analysis

To observe the health status of the ducklings, they were monitored 6 times a day, science they were sent to the laboratory animal room. As DVH induced by DHAV-1 is an extremely acute disease, so duckling will die quickly once the typical clinical symptoms such as opisthotonos and convulsions occurs. For the purpose of ameliorating the suffering of the dying ducklings, CO_2_ euthanasia which was an effective and widely used method was applied. Meanwhile, the dead ducklings were dissected immediately to exclude the numbers of the ducklings died of other disease. During the entire monitoring time, no ducklings died as a result of opithotonos or convulsions and all the euthanized ducklings were autopsied and identified with typical pathological changes of DVH. Along with each monitoring process, the dead duckling numbers in each group were recorded. All dead ducklings were executed and disposed of in bio-safety containers in accordance with local standard protocols. The mortality rate in each group was calculated according to the formula: mortality rate (%) = the number of dead ducklings / the number in the sample group × 100%.

### Histopathological evaluation

The liver tissues were randomly isolated from 5 feathers collected from birds in each group at the acute phase (4 hpi and 8 hpi) (hpi: hours post injection) and stable phase (54 hpi). Theses ducklings (15 feathers per group) were also euthanized by CO_2_ and they were not included in the survival rate. A portion of each isolated liver tissue was instantly fixed in neutral phosphate-buffered 10% formalin solution for 24 h, dehydrated using increasing concentrations of ethanol and embedded in paraffin. Sections of 5 μm of thickness were taken, mounted onto clear, hydrated glass slides, stained with hematoxylin and eosin (H&E) and then examined under a light microscope (Olympus, Japan).

### Hepatic oxidative stress examination

The liver tissues were dissected quickly, placed on ice, weighted and immediately homogenized in ice cold 10 mM Tris-HCl (pH = 7.4). Then, homogenates were centrifuged at 2500 g for 10 min and supernatants were collected in corresponding tubes and stored in—70°C for posterior hepatic SOD, CAT and GPX activities and MDA content examination.

### Mitochondrial ultrastructure analysis

The liver mitochondrial ultrastructure damage was analyzed by using TEM. For TEM analysis, liver tissues were prepared as previous described [25]. In brief, livers were harvested and dissected on ice. Specimens for TEM were fixed in 2.5% glutaraldehyde at 4°C for 24 h, washed with 0.1 M phosphate buffer and post-fixed in osmium tetroxide for 1 h thereafter. Then, samples were washed in cacodylate buffer (0.1 mol/L, pH = 7.4), incubated in uranyl acetate, dehydrated with ascending grades of ethanol concentrations, and finally embedded in araldite CY212. Ultrathin sections (60–70 nm) were cut and stained with lead citrate and uranium acetate prior to visualization under a Tecnai 12 TEM (Philips, Holland).

### Examination of the ATP content in liver tissues

The ATP levels in liver tissues were measured using an ATP assay kit, according to the manufacturer’s protocol. Briefly, tissues were grinded with 200 μL lysis buffer and centrifuged at 12000 g for 5 min. Then, 100 μL supernatant per sample was assessed with 100 μL ATP detection buffer. Luminescence was determined by a GloMax ^™^ 96 microplate luminometer (Promega, America).

### Liver mitochondria isolation and peroxidation injury examination

Livers were rapidly dissected then further processed to isolate mitochondria by using Tissue Mitochondria Isolation Kit according to the manufacturer’s protocol. In brief, livers were isolated from the ducklings and washed with ice-cold PBS. Then 100 mg liver tissue was cut into small cubes with scissors in 1 mL ice-cold mitochondria isolation reagent and homogenized with a handhold homogenizer. After the liver tissues were homogenized, the homogenate was centrifuged at 600 g for 5 min at 4°C. Then the resulting supernatants were decanted and centrifuged at 1100 g for 10 min at 4°C. The mitochondria were finally collected in the sediments and then suspended in mitochondria stock buffer.

The collected mitochondria were lysed with mitochondria lysis buffer and the protein concentrations were determined using BCA protein assay kit. Then the mitochondria solutions were diluted into 0.5 mg protein/mL and used for following mitochondrial SOD, GPX activities and MDA content tests.

## Statistical analysis

All the data were analyzed by IBM SPSS statistics 20.0 sorftware. The data obtained were presented as means ± standard error (S.E.). Duncan’s multiple range test was used to analyze the difference among groups and χ ^2^ test was used to analyze the difference of the mortality rate among groups. *P* values < 0.05 were considered as statistically significant.

## Results

### Clinical curative effect

The clinical curative effect of HRS prescription is presented in Table 1. In the VC group, the mortality rate (71.1%) and the mortality (32 feathers) were both the highest, significantly higher than those of the BC group (*p* < 0.05). Compared with the VC group, ducklings in the HRS group, the mortality rate (48.9%) was significantly lower than that of the VC group (*p* < 0.05) and decreased approximately 22.2%.

**Table 1:** Results of mortality rate of each group.

Group	Samples (feathers)	Final deaths (feathers)	Mortality rate (%)
BC	45	0	0^c^
VC	45	32	71.1^a^
HRS	45	22	48.9^b^

180 six-day-old cherry valley ducklings without inoculated by DHV vaccine were randomly divided into 3 groups: Blank control (BC) group (separately reared), Virus control (VC) group and HRS group. For ducklings in VC and HRS groups, they were challenged with 0.2 mL of DHAV-1 solution by intramuscular injection. The ducklings in the BC group were simultaneously injected with an equal volume of physiological saline. Two hours later, ducklings in the HRS group were orally administrated with prepared drug solution by drinking water, once a day for five days. Finally, the mortality rate in each group was calculated.

^a−c^ Data within a column without the same superscripts differ significantly (*p* < 0.05).

### Changes of hepatic pathologic

**Fig 1:**
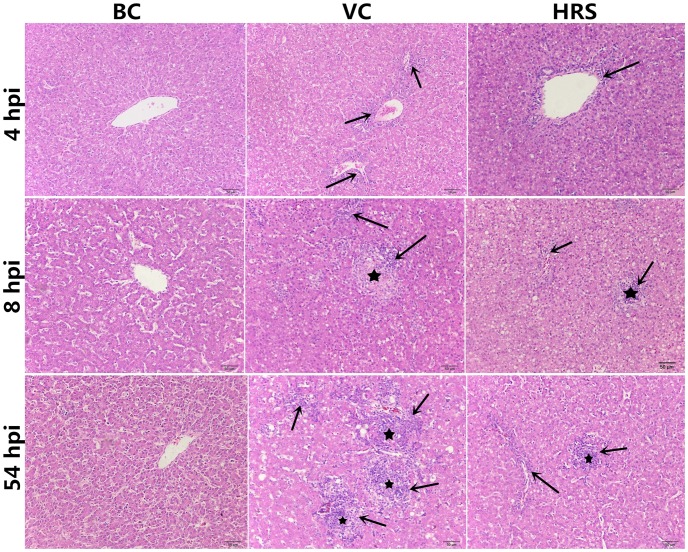
Liver histological changes of each group at 4 hpi, 8 hpi and 54 hpi (hpi: Hours Post-Injection) (HE stain, × 200). Liver tissues were randomly isolated from 5 feathers collected from birds in each group at 4 hpi, 8 hpi and 54 hpi. A portion of each isolated liver tissue was instantly fixed in neutral phosphate-buffered 10% formalin solution and stained with hematoxylin and eosin (H&E). Arrows indicate inflammatory cells and pentagrams indicate necrosis foci.

The liver histological changes of each group at different time points observed by HE staining are summarized in Fig 1. As shown in the figures, no lesion was observed in the liver cross-sections of the BC group at 4 hpi, 8 hpi and 54 hpi. However, the histopathology of the ducklings in the VC and HRS groups showed that a few periportal inflammatory cells (indicated by arrows) were observed at 4 hpi. Eight hours after challenge, an increasing number of inflammatory cells and a few necrosis foci (indicated by pentagrams) were observed in the liver of the VC group. With the development of the disease, the number of the inflammatory cells and the necrosis foci kept increasing, and the necrosis foci areas got much larger in the liver of the VC group at 54 hpi. In contrast, liver histological changes of the HRS group were significantly alleviated with an obvious decrease in inflammatory cell infiltration and necrosis foci areas.

### Changes of hepatic peroxidation indexes

**Fig 2:**
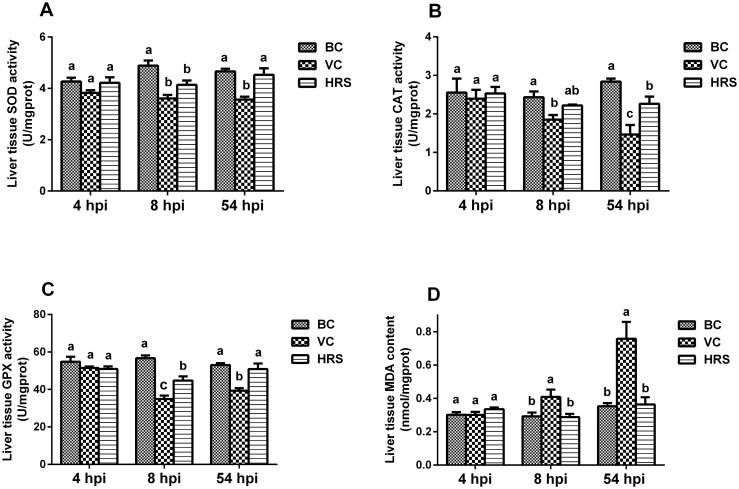
Changes of hepatic peroxidation indexes in liver tissue at 4 hpi, 8 hpi and 54 hpi (hpi: Hours Post-Injection). Liver tissue samples were obtained from individual ducklings of each group at the indicated time points, and the liver tissue SOD (A), CAT (B), GPX (C) activities and MDA (D) content were detected with corresponding assay kit. a-c Bars in the same index at the same time point without the same superscripts differ significantly (*p* < 0.05).

The results of the hepatic peroxidation injury examination are presented in Fig 2. The SOD, CAT, GPX activities and MDA content in the liver tissue homogenate showed no significant difference among the three groups at 4 hpi (*p* > 0.05). However, ducklings in the VC group after challenged for 8 hours, the indexes of SOD, CAT and GPX in the liver tissue homogenate were significantly decreased but the level of MDA was significantly increased compared with those of the BC group (*p* < 0.05). Meanwhile, a similar changing trend was also observed in the HRS group. As for the levels of the SOD and GPX of the HRS group, they were both significantly lower than those of the BC group (*p* < 0.05), whereas they were elevated a lot compared with the VC group and the difference of the GPX activity showed statistically significant (*p* < 0.05). The MDA content in liver tissue homogenate of the HRS group changed very little compared with that of the BC group, and it showed significantly lower than that of the VC group (*p* < 0.05). Ducklings of the VC group at 54 hpi, the levels of the SOD, CAT and GPX in liver tissue homogenate were still much lower and the content of the MDA was much higher than those of the BC group, and significant differences were observed (*p* < 0.05). In contrast, almost all the indexes of the HRS group were return to the normal level, and they all were significantly higher (SOD, CAT and GPX activities) or lower (MDA content) than that of the VC group (*p* < 0.05).

### Changes of ATP content in liver tissue homogenate

**Fig 3:**
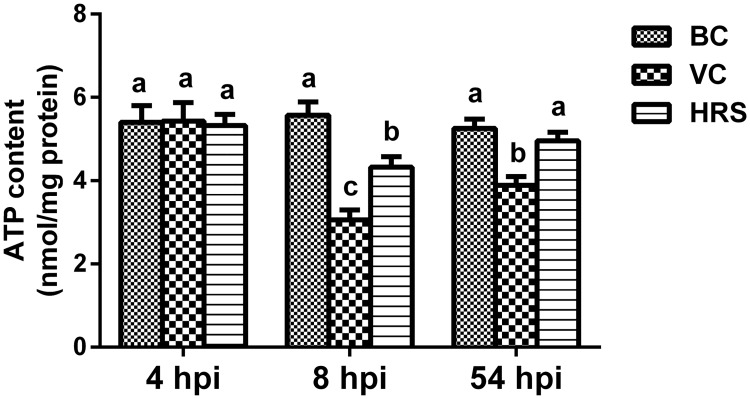
Changes of ATP content in liver tissue homogenate at 4 hpi, 8 hpi and 54 hpi (hpi: Hours Post-Injection). Liver tissue samples were obtained from individual ducklings of each group at the indicated time points, and the ATP content in liver tissue was detected with ATP assay kit. a-c Bars in the same index at the same time point without the same superscripts differ significantly (*p* < 0.05).

The results of the ATP content in liver tissue homogenate of each group are showed in Fig 3. At 4 hpi, the content of ATP in liver homogenate of each group was similar, and no significant difference was observed among these groups (*p* < 0.05). However, the ATP contents in liver tissue homogenate of the VC and HRS groups were both significantly decreased compared with that of the BC group at 8 hpi (*p* < 0.05). At the same time, the ATP content in liver tissue homogenate of the ducklings after administrated with HRS was significantly elevated compared with that of the VC group (*p* < 0.05). Ducklings after challenged with DHAV-1 for 54 hours, the ATP content in liver tissue homogenate was still significantly lower than that of the BC group (*p* < 0.05). On the contrary, although the ATP content in liver tissue homogenate of the HRS group was still a slight lower than that of the BC group, there was no significant difference between these two groups (*p* > 0.05). Moreover, the ATP content in liver tissue homogenate of ducklings treated with HRS was significantly elevated compared with that of the VC group (*p* < 0.05).

### Changes of mitochondrial ultrastructure

**Fig 4:**
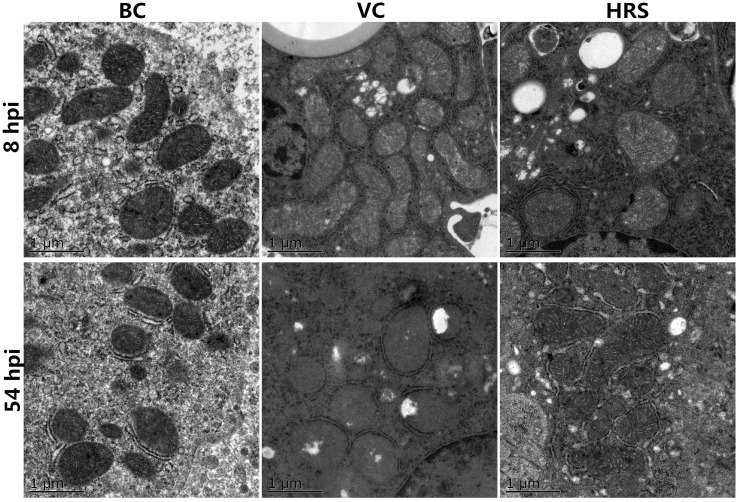
Changes of mitochondria ultrastructure at 8 hpi and 54 hpi (hpi: Hours Post-Injection). Liver tissue samples were obtained from individual ducklings of each group at the indicated time points, and the ultrastructure changes of liver mitochondria were analyzed by using TEM. Scale bar, 1 μm.

The results of the mitochondrial ultrastructure of each group are showed in Fig 4. As no difference was observed among BC, VC and HRS groups at 4 hpi, therefore we did not present these results here. For the BC group, the mitochondria showed normal shape and distribution of cristae and intact architecture at 8 hpi and 54 hpi. However, the mitochondrial ultrastructure examination results of the VC group showed swollen mitochondria and the reduction of the cristae quantity at 8 hpi. Compared to the VC group, the lesion degree of mitochondrial ultrastructure of the HRS group was alleviated a lot, such as, the increasing number of the cristae and the decreasing of the swollen degree. At 54 hpi, rarefaction and vacuoles of the mitochondrial ultrastructure, disappearance of the mitochondrial cristae was observed in the VC group. In contrast, the mitochondrial ultrastructure of the HRS group was much more clear and intact than that of the VC group. Furthermore, the number and the arrangement of the mitochondrial cristae were much more close to the BC group.

### Changes of the mitochondrial peroxidation injury indexes

**Fig 5:**
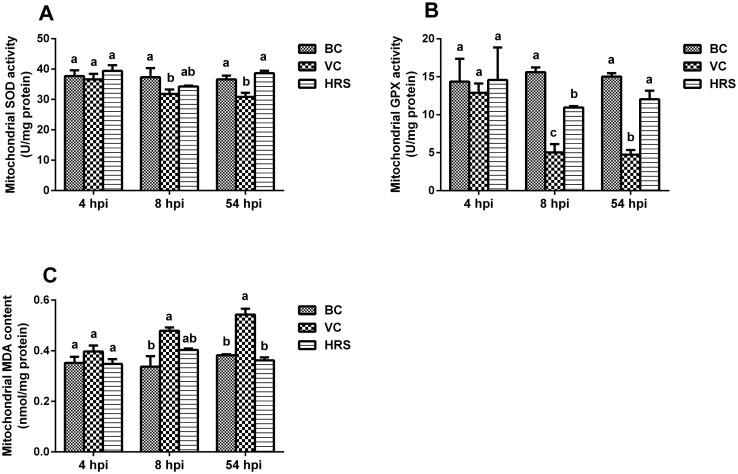
Changes of the mitochondrial peroxidation injury indexes at 4 hpi, 8hpi and 54 hpi (hpi: Hours Post-Injection). Liver tissue samples were obtained from individual ducklings of each group at the indicated time points to isolate mitochondria. Then the mitochondrial SOD (A), GPX (B) activities and MDA (C) content were detected with corresponding assay kit. a-c Bars in the same index at the same time point without the same superscripts differ significantly (*p* < 0.05).

The results of the mitochondrial SOD, GPX activities and MDA content of each group are presented in Fig 5. At 4 hpi, all the indexes among the BC, VC and HRS groups were at same level and no significant difference was observed (*p* > 0.05). At 8 hpi, the mitochondrial SOD and GPX activities were significantly decreased (*p* < 0.05), and the MDA content was significantly increased compared with those of the BC group (*p* < 0.05). However, at this moment, only the mitochondrial GPX activity of the HRS group significantly decreased compared with that of the BC group (*p* < 0.05), and was significantly higher than that of the VC group (*p* < 0.05). At 54 hpi, the significant differences of all the indexes between the VC and BC groups were observed (*p* < 0.05). In contrast, all the indexes of the HRS group were return to almost normal level, the activities of mitochondrial SOD and GPX were significantly higher than those of the VC group (*p* < 0.05), and the mitochondrial MDA level was significantly lower than that of the VC group (*p* < 0.05).

## Discussion

Comparison of mortality rate can directly reflect the curative effect of a drug. In this study, we evaluated the curative effective of HRS, and the results showed that the mortality rate of the ducklings infected with DHAV-1 could be significantly decreased after treated with HRS (Table 1). Combined with our previous research *in vivo* [13], these results suggest that the curative effect of HRS is stable and reliable.

Facing with the invasion of the pathogens such as viruses, bacteria and fungi, host has developed defensive immune systems. In vertebrates, two complementary immune systems (innate immune system and adaptive immune system) are available to recognize and eliminate pathogens [26]. As the first acting arm, innate immune system can recognize various kinds of pathogens by pattern recognition receptors (PRRs) [27]. Consequently, once the pathogens invade into the host body, the innate immune system will be activated and initiate the inflammatory response within a short period. Among these inflammatory cells, phagocytes such as neutrophils and mononuclear phagocytes can also be recruited and produce ROS to kill the pathogens during the so-called “respiration burst” [28]. However, ROS are not just helpful weapons of phagocytes but are harmful agents causing oxidative stress and damage nearby cells and molecules at exorbitant concentration [29].

In our research, we also observed the inflammatory cell infiltration as early as 4 hpi (Fig 1). It indicates that the inflammatory response was triggered and a number of ROS have been produced to against DHAV-1. Interestingly, most of the inflammatory cells appeared around the blood vessel (Fig 1). It may indicate that DHAV-1 was mainly transported by blood circulation after invading into the host body. Compared with the VC group, the quantity of inflammatory cells of the HRS group was almost at the same level at 4 hpi (Fig 1). It suggests that HRS cannot enhance the recruitment of the inflammatory at this emergency period. Although a number of ROS were generated along with inflammatory infiltration at this moment, we did not detect the oxidative stress in the liver tissues (Fig 2). It may suggest that the antioxidant system could effectively eliminate the excessive ROS to keep a relative balance status of the free radical metabolism. When ducklings were challenged with DHAV-1 for 8 hours, the histological changes of the VC group were aggravated with the appearance of the necrosis foci and the increasing number of the inflammatory cells (Fig 1). At the same time, the activities of liver tissue SOD, CAT and GPX were significantly decreased compared with those of the BC group (Fig 2). For SOD, it degrades superoxide radical into hydrogen peroxide. CAT and GPX degrade hydrogen peroxide into H_2_O. They work together to scavenge ROS. What’ more, as the main evaluation index of lipid peroxidation injury [30], the level of the liver tissue MDA was also significantly elevated compared with that of the BC group (Fig 2). These results may suggest that with the excessive on-going release of the ROS by “respiratory burst”, the free radical overwhelmed the antioxidant defenses and disturbed the metabolism balance thus causing the hepatic injury. At 54 hpi, the hepatic injury further aggravated with the increasing number of inflammatory cells and the expansion of the necrosis foci (Fig 1), the activities of all the anti-oxidative enzymes were at rather low levels (Fig 2), and the level of MDA was further elevated (Fig 2). The decrease of these anti-oxidative enzyme levels may be caused by the depletion during the excessive ROS scavenging process. Therefore, the levels of these anti-oxidative enzymes can reflect not only the anti-oxidative activity, but also the ROS level. These results indicate that the autologous restoration function could not effectively eliminate the ROS hence resulting in a bad prognosis in the VC group. However, for infected ducklings treated with HRS, the histological changes were alleviated greatly (Fig 1), and all the peroxidation injury evaluation indexes finally bounced back to close to normal levels (Fig 2). These results suggest that HRS possesses antioxidant activity in liver tissue to decrease the oxidative stress in liver so that exerts its hepatoprotective effect.

As is well known, liver is the central organ of the body’s energy metabolism and conversion [31]. Meanwhile, hepatocytes are highly dependent on ATP due to their vast energy demand [32, 33]. Mitochondria are well known as the “power house” of the cells, so they play crucial roles in maintaining cell normal function. However, mitochondria are the most sensitive organelles to oxidative stress for their rich in polyunsaturated fatty acids, so they are extremely vulnerable to free radicals [20]. Although mitochondria possess their own antioxidant systems such as SOD and GPX, which were the dominating defenders against oxidative injury, the excessive ROS can overwhelm these defenses [34, 35]. What’s worse, defective mitochondria are another potential source of ROS, which can aggravate the oxidative stress [36]. Convincing evidence demonstrated that many oxidative stress diseases were accompanied with mitochondrial dysfunction [37]. As mentioned above, the oxidative stress caused by DHAV-1 in liver tissue has been firstly discovered (Fig 2), but whether the mitochondrial function can be perturbed or not is unknown. Therefore, we performed the posterior research to investigate it.

ATP is the production of the oxidative phosphorylation and mainly responsible for most of the body needed energy [38]. The level of ATP can directly reflect the energy state of the cells and the mitochondrial function [39]. Researches have demonstrated that mitochondrial dysfunction was always accompanied with ATP depletion [37]. In the present study, we found that ATP content in liver tissue showed no significant difference among the three groups at 4 hpi (Fig 3). It indicates that the supply of the energy was not affected, and the function of the mitochondria kept a normal status at the initial period of virus invasion. It is consistent with our TEM analysis results (Fig 4) by which the mitochondrial ultrastructure lesion can be visually observed at this time point. Meanwhile the mitochondrial peroxidation injury evaluation results showed that no significant difference was observed among the three groups in all the indexes. One possible explanation of the above results is that the antioxidant defenses in liver tissue had already effectively eliminated the excessive ROS (Fig 2) thus avoiding the invasion and the influence on the mitochondria. At 8 hpi, with the accumulation of the ROS in the liver tissue, the antioxidant defenses were disturbed with the decline of the antioxidant enzyme activities (Fig 2) leading to the injury in the liver (Fig 1). Just then, we also observed the decline of the ATP content in the liver tissue (Fig 3), the ultrastructure lesion of the mitochondria (Fig 4), the decrease of the antioxidant enzyme activities and the increase of the MDA level in the mitochondria (Fig 5). These results indicate that the outer antioxidant defense barrier of the mitochondria was torn thus causing the attack of the excessive ROS to the mitochondria, and finally led to the mitochondrial oxidative stress and the ultrastructure lesion. As described above, mitochondria are the major source of the ROS within cells, so mitochondrial dysfunction will exacerbate the oxidative stress in turn thereby forming a vicious cycle. Moreover, an increasing number of researches demonstrate that mitochondrial oxidative stress can mediate cell death not only via apoptosis but also via necrosis [40, 41]. Therefore, it is not difficult to explain the further aggravation of the hepatic injury and the mitochondria dysfunction in the VC group at 54 hpi. In contrast, based on the antioxidant activity of HRS, the attack of the ROS to the mitochondria was attenuated both at 8 hpi and 54 hpi, thus reducing the release of the ROS from the mitochondria and alleviating the hepatic and the mitochondrial injury.

## Conclusion

As the major target organelle of ROS, mitochondrial dysfunction is involved in many kinds of oxidative stress disease. In this research, we discovered the oxidative stress in the liver tissue during the pathological process of DVH induced by DHAV-1, and further study confirmed the existence of mitochondria dysfunction. Ducklings infected with DHAV-1 after treated with HRS, the hepatic injury and the mitochondrial dysfunction were greatly alleviated. All these data may suggest that HRS can effectively suppress the oxidative stress in liver tissue thus attenuating the ROS attack to the mitochondria then exerting its mitochondria protective effect.
